# Immune consequences of penfluridol treatment associated with inhibition of glioblastoma tumor growth

**DOI:** 10.18632/oncotarget.17425

**Published:** 2017-04-26

**Authors:** Alok Ranjan, Stephen Wright, Sanjay K Srivastava

**Affiliations:** ^1^ Department of Biomedical Sciences and Cancer Biology Center, Texas Tech University Health Sciences Center, Amarillo, TX 79106, USA; ^2^ Departments of Internal Medicine and Biomedical Sciences, Texas Tech University Health Sciences Center, Amarillo, TX 79106, USA; ^3^ Department of Immunotherapeutics and Biotechnology, Texas Tech University Health Sciences Center, Abilene, TX 79106, USA

**Keywords:** MDSC, glioblastoma, anti-psychotic drug, Treg, macrophages

## Abstract

Glioblastoma is the most common and lethal brain tumor associated with only 12% median survival rate of patients. Despite the development of advanced surgical, radiation or use of combinations of anti-cancer drugs, treatment for glioblastoma patients is still a challenge. The major contributing factor in glioblastoma progression and resistive nature is its ability to evade the immune surveillance. Hence, modulating the immune system in glioblastoma tumors could be an important strategy for anticancer therapeutics. Penfluridol, an antipsychotic drug has been shown to have anti-cancer properties in our recently published studies. The present study evaluates the immune response of penfluridol in glioblastoma tumors. Our results demonstrated that penfluridol treatment significantly suppressed glioblastoma tumor growth. Our current results demonstrated about 72% suppression of myeloid derived suppressor cells (MDSCs) with penfluridol treatment in mouse bearing U87MG glioblastoma tumors. MDSCs are known to increase regulatory T cells (Treg), which are immunosuppressive in nature and suppresses M1 macrophages that are tumor suppressive in nature. Our results also showed suppression of regulatory T cells as well as elevation of M1 macrophages with penfluridol treatment by 58% and 57% respectively. Decrease in CCL4 as well as IFNγ with penfluridol treatment was also observed indicating decrease in overall tumor inflammation. This is the first report demonstrating immune modulations by penfluridol treatment associated with glioblastoma tumor growth suppression prompting further investigation to establish penfluridol as a treatment option for glioblastoma patients.

## INTRODUCTION

Glioblastoma (GBM) is a tumor that arises from astrocytes [1]. Astrocytes are star shaped cells and are known to support brain tissue. Glioblastoma tumors are highly malignant and aggressive in nature because cells divide quickly and are supported by a large network of blood vessels providing nutrients [1]. Malignant glioblastoma is highly infiltrated by myeloid-derived suppressor cells (MDSCs), which play a critical role in glioblastoma tumor progression and metastasis [2–4]. MDSCs are a heterogeneous group of cells and are a major component of tumor microenvironment. MDSCs, generated in bone marrow and in host bearing tumors, migrate to lymphoid organs and tumors, and support the formation of tumor microenvironment [5]. Acquired resistance to chemotherapy is difficult to overcome during cancer treatment. Induction and expansion of MDSCs play a critical role in chemoresistance [6]. Currently, glioblastoma is difficult to manage by conventional chemotherapies. Hence, new treatment option for glioblastoma is indispensable.

Like MDSCs, regulatory T cells (Treg) are also a part of immunosuppressive tumor microenvironment [7]. The association of regulatory T cells in overall poor prognosis of several neoplasms has been established [8]. In addition, recent studies demonstrated that MDSCs can also induce regulatory T cells [9]. On the other hand, macrophages have critical role in cancer progression, and play a key role in inflammation and cancer [10]. Tumor-associated macrophages facilitate neoplastic transformation as well as promote signaling cascades for metastasis [11]. MDSCs and macrophages are present in most solid tumors and play a critical role in overall inflammation and immunosuppressive activity [12]. Cross-talk between MDSCs and macrophages promote tumor prognosis by affecting anti-tumor immunity [12]. The resulting chronic inflammation can predispose to cancer [13]. Several anti-inflammatory drugs have been shown to have an anti-cancer effect [14]. Interestingly, Baune et.al has demonstrated in a study that antipsychotic drugs have an anti-inflammatory effect [15].

Penfluridol, an antipsychotic drug has been demonstrated by us to inhibit the growth of metastatic breast tumors in brain [16]. In another study, we observed that penfluridol significantly suppresses the growth of various glioblastoma tumor cells *in vitro* and *in vivo* [17]. In the current study, we evaluated immune consequences of penfluridol treatment associated with inhibiting glioblastoma tumor growth.

Our results show substantial growth suppression of glioblastoma tumors and MDSC levels by penfluridol treatment. In addition, penfluridol treatment reduced Tregs and enhanced M1 macrophages. This is the first report of immune modulation by penfluridol treatment associated with suppression of glioblastoma tumor growth.

## RESULTS

### Penfluridol inhibits glioblastoma tumor growth

**Figure 1:**
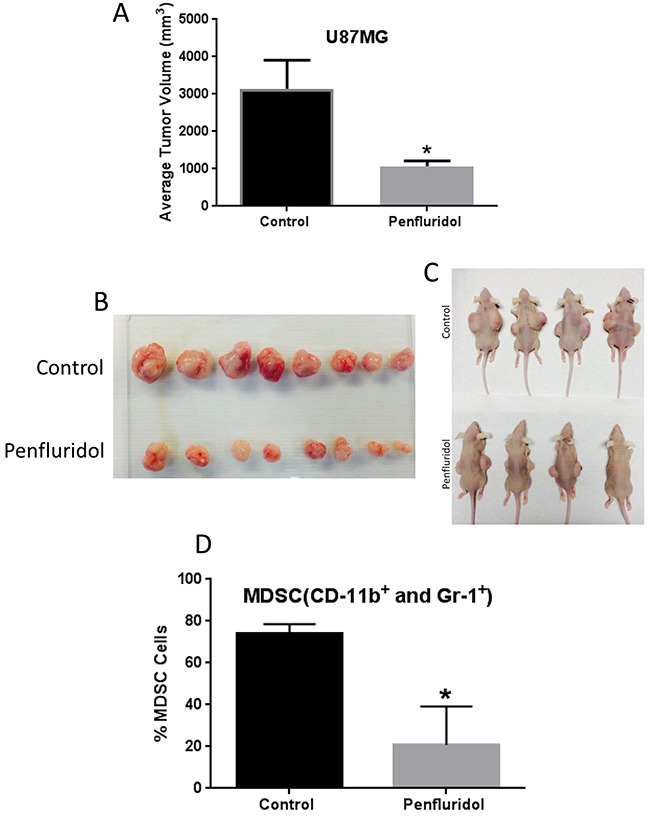
Penfluridol suppresses glioblastoma tumor growth and associated MDSCs 1×10^6^ U87MG cells in 1:1 mixture of PBS and matrigel were implanted in right and left flanks of 4-6 week old athymic nude mice. Treatment with 10mg/kg penfluridol by oral gavage everyday started once tumor size was 70-100mm^3^ after tumor cells injection till day 48. Tumors from all control and penfluridol treated mice were removed at day 48. **(A)** Bar graph representing average tumor volume of control and penfluridol treated mice at day 48. Values were plotted as mean ± SEM. **(B-C)** Representative images of tumors as well as mice bearing tumors from control and treated group. Peripheral blood mononuclear cells (PBMCs) were collected from the blood obtained from control and penfluridol treated U87MG tumor-bearing athymic nude mice. Modulation of CD11b^+^ and Gr-1^+^ cells was analyzed by immunostaining and fluorescence cytometry to determine the effects of penfluridol on mouse myeloid derived suppressor cells (MDSCs); statistical analysis was performed by Student's t test. **(D)** Bar chart showing percent MDSCs which were double stained with CD11b and Gr-1 mouse antibodies. *Statistically significant at p<0.05 when compared with control.

To determine the efficacy of penfluridol in inhibiting the growth of glioblastoma, U87MG glioblastoma cells were implanted subcutaneously on both flanks of athymic nude mice. Once each mouse attained about 70-100mm^3^ tumors, mice were randomly divided into two groups. Control mice received vehicle only whereas the treatment group of mice received 10mg/kg penfluridol everyday by oral gavage. The experiment was terminated at day 48 due to excessive tumor burden in control mice. After 48 days of penfluridol treatment, glioblastoma tumor growth was suppressed by 65% (Figure 1A-1C).

### Reduction in myeloid-derived suppressor cells with penfluridol treatment

Myeloid-derived suppressor cells (MDSCs) are known to promote growth of tumors. High infiltrations of MDSCs are also known to regulate stem cells like characteristics in tumors. We used CD11b and Gr1 markers to analyze mouse MDSCs after treatment of mice with 10mg/kg penfluridol by oral gavage everyday till day 48. Blood from control and penfluridol treated mice was collected and PBMCs were separated. MDSCs were analyzed by gating on forward and side scattered profile of the cells using flow cytometer. We observed a 72% reduction of mouse MDSCs with penfluridol treatment (Figure 1D). This observation indicated that penfluridol treatment suppressed MDSCs, which play a significant role in promoting tumor growth.

### Modulation in spleen weight with penfluridol treatment

**Figure 2:**
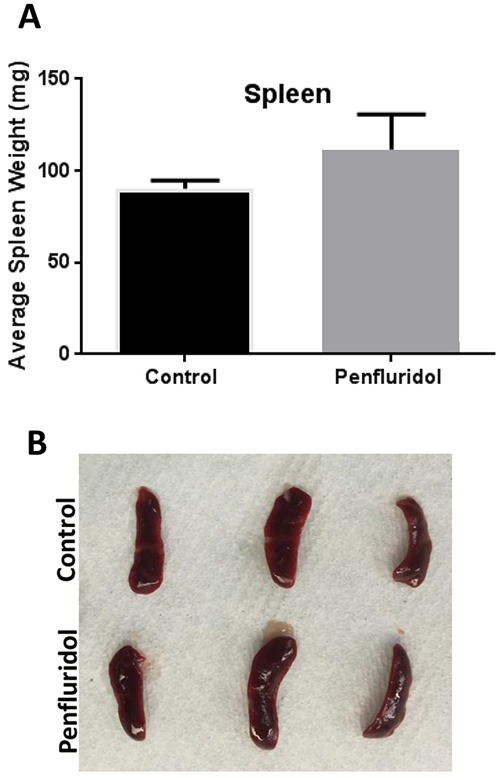
Increase in spleen weight with penfluridol treatment: Spleens from control and penfluridol treated mice were removed and weights of spleens were taken **(A)** Bar chart showing average weight of spleen in control and penfluridol treated group. Values were plotted as mean ± SEM. **(B)** Image of spleen from control and penfluridol treated mice.

The spleen is a peripheral immune organ and plays a critical role in regulating the immune system of the body. Shrinkage of spleen as well as inhibition of spleen cell proliferation has been observed in late stage tumors where the immune system is suppressed [18, 19]. Our results showed that the average weight of spleens in mice treated with penfluridol was modestly increased as compared to the spleens from control mice (Figure 2A-2B), indicating that penfluridol may have increased splenic cells proliferation, which correlate with inhibition of tumor growth associated with immune system.

### Suppression of regulatory T cells by penfluridol treatment

**Figure 3:**
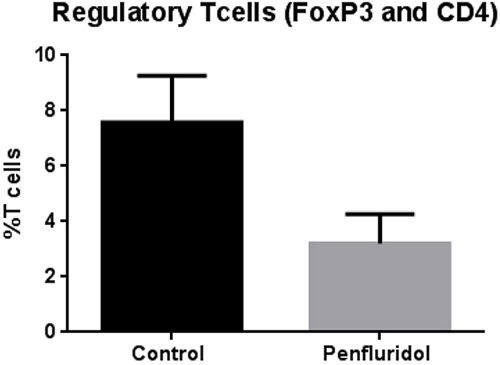
Penfluridol suppresses regulatory T cells (Treg) About 40 × 10^6^ Human peripheral blood mononuclear cells (PBMCs) cells were injected i.p. in 100 μL PBS in SCID-NOD mice. The treatment group received 10mg/kg penfluridol by oral gavage everyday; control mice received vehicle alone. Modulation of regulatory T (Treg) cells was monitored by immunostaining and fluorescence cytometry to determine the effects of penfluridol. Values were plotted as mean ± SEM. Statistical analysis was performed by Student's t test.

Translation of mouse data to human fails due to significant differences in mouse and human immune systems. In order to confirm effects of penfluridol treatment on human immune system, we injected 40 × 10^6^ human PBMCs (isolated from human blood) intraperitoneally in SCID-NOD mice. A week after human PBMC injection, each mouse was injected with 1 × 10^6^ U87MG glioblastoma cells subcutaneously. Penfluridol 10mg/kg was administered to mice everyday by oral gavage once tumor volume reached around 70-100 mm^3^, whereas the control group of mice received vehicle only. The experiment was terminated at day 40 due to excessive tumor burden in the control group of mice. After termination of the experiment, PBMCs were collected from the blood of control and penfluridol treated mice and double stained with FoxP3 and CD4 antibodies for regulatory T cells (Treg). Our results show that penfluridol treatment reduced regulatory T cells (Treg) by 58%, indicating an increase in immune surveillance in mice bearing human tumors (Figure 3).

### Elevation of M1 macrophages with penfluridol treatment in the tumor microenvironment

**Figure 4:**
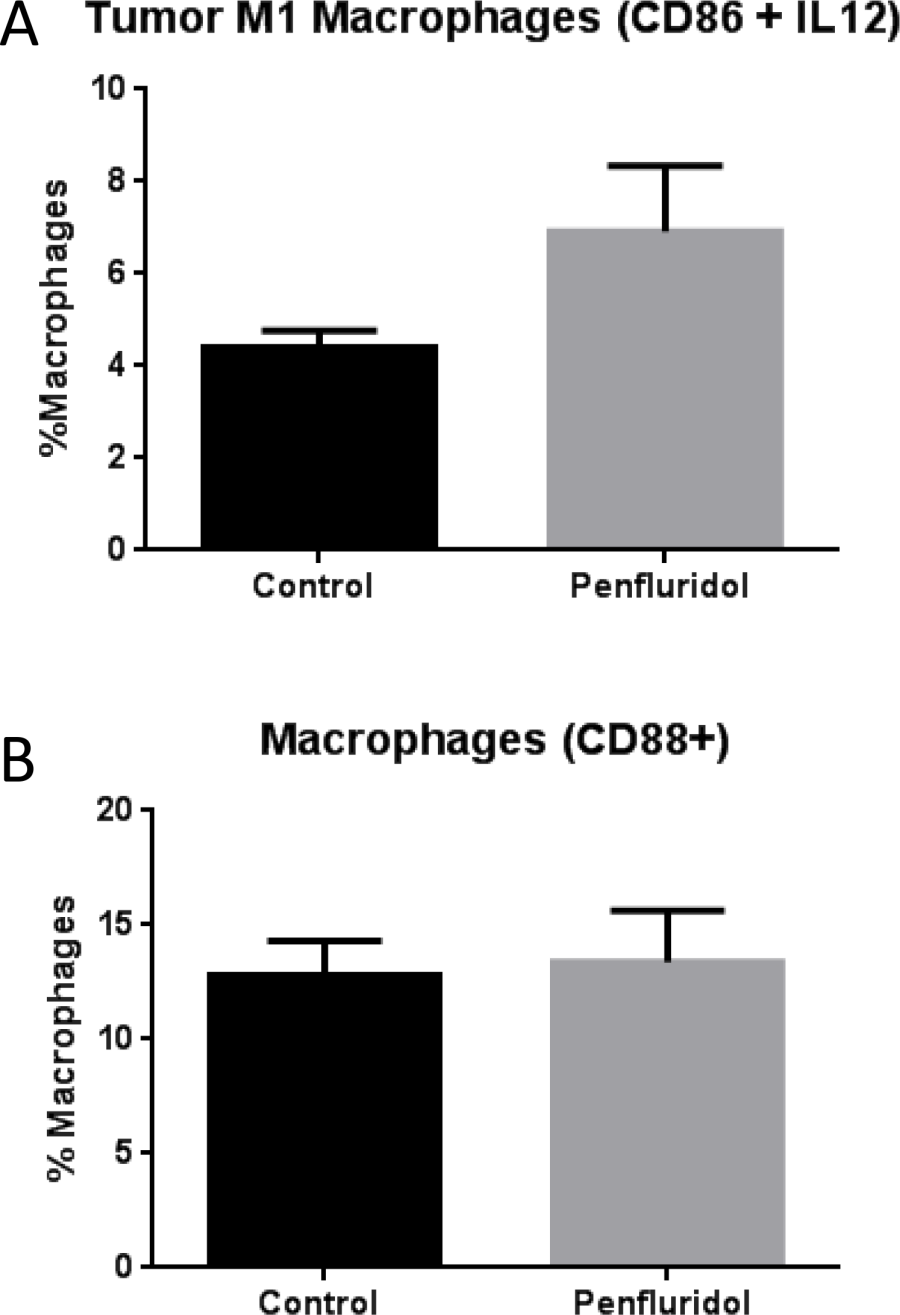
Elevation of M1 macrophages in tumor with penfluridol treatment Subcutaneously implanted U87MG glioblastoma tumors in SCID-NOD mice were removed from control and penfluridol treated mice at day 40. Tumors were processed and suspended into single cell suspension with the help of tumor dissociator kit and use of gentle MACS dissociator. Effect of penfluridol treatment on macrophages was analyzed by immunostaining and florescence cytometry. **(A)** Bar graph representing percent monocytic cells double stained with CD86 and IL12 human specific antibodies. **(B)** Percent cells which were single stained with CD88 human specific antibody.

Tumors from control and penfluridol treated mice were aseptically removed after terminating the experiment at day 40. Tumors were suspended into single cell suspension using a tumor dissociation kit and gentle MACS Dissociator. Single cell suspension from control and penfluridol treated tumors were processed and analyzed after double staining with CD86 and IL12 antibodies for antigens present on M1 macrophages. M1 macrophages are known to be involved in tumor growth suppression [20]. Our results demonstrated that penfluridol treatment resulted in a 57% increase of M1 macrophages as compared to control tumors (Figure 4A). Interestingly, we did not observe any change in the cells stained with CD88 expression, a marker of macrophages [21].

### Penfluridol suppressed tumor inflammation

**Figure 5:**
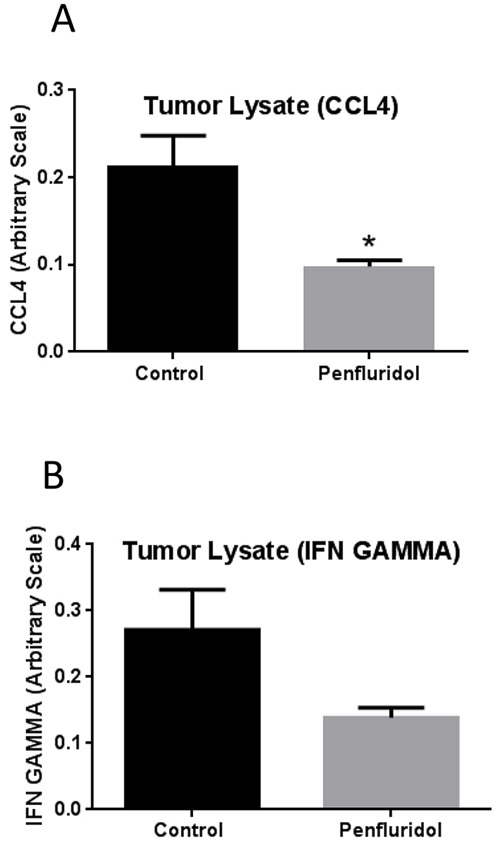
Suppression of tumor inflammation with penfluridol treatment After removing U87MG tumors, a part of the tumors were lysed and protein concentration was estimated in different control and penfluridol treated tumor lysate. Equal amount of protein was used to perform ELISA assay for CCL4 and IFNγ. **(A-B)** Bar graph representing CCL4 and IFNγ levels in the tumors from control and treatment group. Values were plotted as mean ± SEM. *Statistically significant at p<0.05 when compared with control.

As chronic inflammation plays a significant role in tumor progression, we decided to analyze inflammatory markers in the tumor microenvironment after penfluridol treatment. Subcutaneously implanted U87MG tumors from SCID-NOD mice injected with human PBMCs were examined for inflammatory markers after terminating the experiment. Tumors obtained from control and penfluridol treated mice were lysed and ELISA assay was performed for IFNγ and CCL4. IFNγ and CCL4 are known to play critical roles in tumor progression [22, 23]. Our results demonstrated that penfluridol treatment resulted in 54% and 49% reduction of CCL4 and IFNγ respectively, indicating suppression of inflammation in the tumor microenvironment (Figure 5A-5B).

## DISCUSSION

Glioblastoma is one of the deadliest cancers. Currently, radiation and chemotherapy are the available treatments for glioblastoma, however, glioblastoma cells are radioresistant and chemoresistant leading to reoccurrence after conventional therapy [24]. The immune system's natural ability to recognize and kill abnormal cells in the body may prevent development of cancer. However, cancer cells evade immune response by inducing several immune cells that can suppress natural defense of the body [25]. Tumor progression has long been associated with chronic inflammatory microenvironment and infiltration of MDSCs [26]. MDSCs infiltration of the tumor microenvironment has also been associated with glioblastoma stem cells characteristics [27]. In addition, glioblastoma progression is connected with immunosuppression driven by elevation in Treg and MDSCs in the tumor microenvironment [26]. Elevated Treg in peripheral blood and tumor microenvironment has been associated with tumor progression. Temozolomide, an FDA approved drug against glioblastoma has been known to reduce circulating Treg further increasing the activity of cytotoxic T cells against tumors [28, 29]. Furthermore, macrophages are known to be associated with MDSCs and involved in inflammation and immunosuppressive effects in the tumor microenvironment [12]. Overall, glioblastoma tumors create an immunosuppressive environment and engage several pathways to evade innate immune surveillance.

In our previous study, we demonstrated that penfluridol treatment significantly suppressed the growth of metastatic breast tumors in brain [16]. Our results from another study showed that 10mg/kg penfluridol everyday by oral gavage suppressed the growth of glioblastoma tumors in a subcutaneous and intracranial model [17]. In the current study, we evaluated immune modulations after treatment with penfluridol. Several lines of evidence have suggested that chronic inflammation predisposes to cancer [13]. Our results demonstrated that oral administration of 10mg/kg penfluridol everyday for 48 days reduced mouse MDSCs by 72%. Generally, chemotherapy is associated with immune suppression in the patients. Polysaccharides, such as ginseng, when used in combination with anti-cancer drugs was shown to improve drug induced immunosuppression [30]. Beneficial effect of polysaccharide in combination with anti-cancer drugs was associated with increase in spleen weight and stimulation of lymphocyte proliferation [30]. Similarly to these studies, we also observed a minor increase in spleen weight with penfluridol treatment indicating increased splenic activity and immune stimulation, which may have led to enhanced immune surveillance to inhibit tumor growth.

Different animal models are used to mimic human disease, however, in most of the cases, translation of mouse data to human fails due to significant differences between mouse and human immune systems [31]. We therefore evaluated the effect of penfluridol in glioblastoma tumors by injecting human PBMCs in SCID-NOD mice prior to human tumor implantation. MDSCs inhibit adaptive immunity of the body by inducing Treg cells [32]. Treg cells are CD4^+^ T cells with elevated FOXP3 and are known to suppress innate immune responses [33]. Our results showed that penfluridol treatment by oral gavage reduced Treg cells in SCID-NOD mouse injected with human PBMCs. Macrophages, primarily M1 and M2, subtypes are a major source of inflammatory cytokines. M1 macrophages secrete interleukin 12 (IL-12) that helps in generation of T helper 1 (TH-1) adaptive immunity to directly induce cytotoxic effects in tumor cells [34]. M2 macrophages are immune suppressive in nature, contribute to matrix remodeling and hence support tumor growth [35]. M1 macrophages are characterized by high expression of CD86 and IL-12 [36]. In our study, we observed that tumor growth suppression by penfluridol treatment was associated with increased M1 macrophages, which may have led to induction of cytotoxic effect in tumor cells.

Inflammation has long been associated with tumor growth [37]. It plays critical role in modulating immune surveillance and hence altered response to therapy. Infiltrating macrophages have been established as the critical component of inflammation during tumor progression. It has also been established that CCL4 plays a crucial role in macrophage-mediated tumorigenic signaling [22]. In our current study, we observed that penfluridol treatment reduced infiltrating human CCL4 in the tumor microenvironment by 54% in SCID-NOD mice injected with human PBMCs. Under certain conditions, IFNγ plays critical role in anti-tumor host immunity and supports tumor growth [23]. In a few clinical trials, IFNγ treatment negatively affected patients outcome whereas in other trials, it provided positive effect in cancer patients [23]. Thus IFNγ has been established to have dual role: one as a hallmark of anti-tumor immunity and another to support tumor growth by immune escape phenomena, a mechanism mediated through PD-L-1 expression [23]. Interestingly, we observed that penfluridol significantly suppressed human IFNγ in the tumor microenvironment in SCID-NOD mice injected with human PBMCs and implanted with human U87MG glioblastoma tumors.

In summary, suppression of GBM tumor growth correlated with reduction of immune suppressive cells (Treg and MDSC), an increase of tumor killing macrophages (M1), and reduction of markers of chronic inflammation (CCL4 and IFNγ) in the tumor microenvironment. We suggest that immune enhancement may have been involved in inhibition of tumor growth of glioblastoma. This is the first report, demonstrating immune modulation with penfluridol treatment associated with glioblastoma tumor growth inhibition. An imbalance in the immune system during tumor progression is associated with chemoresistant tumors and our study lays the foundation of effective treatment for glioblastoma by immune modulation. Our study further supports future clinical development of immunotherapies to suppress glioblastoma and preclinical investigation of combining temozolomide and penfluridol and other immune modulatory agents to attempt to eliminate glioblastoma.

## MATERIALS AND METHODS

### Ethics statement

Experiments in mice were conducted in accordance with the ethical standards and according to approved protocol by the Institutional Animal Care and Use Committee (IACUC), Texas Tech University Health Sciences Center, Amarillo, Texas.

### Cell culture

U87MG glioblastoma cells used in this study were purchased from ATCC and maintained in EMEM supplemented with 10%FBS and 1% PSN. Cells used in the experiments were within twenty passages after receipts or resuscitation.

### U87MG subcutaneous implantation

4-6 week old female athymic nude mice were purchased from Harlan Laboratories (Livermore, CA). IACUC approved the use of athymic nude mice. All the experiments were performed under the strict compliance and regulations. As described by us previously, 100μL cell suspension containing 1×10^6^ U87MG cells in 1:1 mixture of PBS and matrigel were implanted in both flanks of mice. Mice were divided into two groups with 5 mice in each group once tumor volume reached around 70-100 mm^3^. Group I served as control and received the vehicle only whereas group II received 10mg/kg/day penfluridol by oral gavage. Penfluridol was purchased from Sigma-Aldrich and stock of penfluridol was made in DMSO which was further diluted in water/PEG300/ethanol/2% acetic acid in 8:3:0.13:1 v/v [38]. Tumor volume was measured twice a week till day 48 by using vernier caliper and as described by us before [16, 39]. At day 48, mice were humanely sacrificed and tumors were removed. The results of this experiment have been published by us recently [17]. A part of the tumor from this experiment was used for current study.

### PBMC collection from mouse blood and flow cytometric analysis

To evaluate the effect of penfluridol treatment on immune cells, blood was collected from control and treatment group. The blood collected from each mouse by cardiac puncture was diluted 10 fold with RBC lysis buffer and incubated for 15 minutes at room temperature on a shaker. RBC lysis step was repeated in case complete lysis not achieved. After lysis of RBCs, cells were washed with PBS followed by a second washing with FACS buffer (2% heat inactivated fetal bovine serum and 2 mM ethylenediaminetetraacetic acid (EDTA) in PBS) as described before by us [40]. PBMC from each mouse were equally divided into two groups. Each set of cells was re-suspended in an equal volume of FACS buffer. CD11b Gr-1 FoxP3, CD4, CD86, IL12 and CD88 antibodies were purchased from Miltenyi Biotech. The samples were first blocked using blocking reagent for 15 minutes at 4°C in dark. After blocking, cells were washed and suspended in FACS buffer. After suspension in FACS buffer, appropriate antibodies were added and samples were incubated for 30 minutes on ice in dark. The samples were then washed and re-suspended in 300μL of FACS buffer. Accuri C6 flow cytometer software was used to analyze the effect of penfluridol treatment on MDSCs, regulatory T cells (Treg) and macrophages.

### Human PBMC isolation from buffy coat

Healthy human buffy cones from anonymous donors were obtained from Coffee Memorial Blood Bank, Amarillo, TX. Samples from each buffy cone were diluted with PBS to make final volume of 50ml. Diluted samples were divided into two tubes with 25ml each, after which, 15ml PBS was further added. Samples were gently layered over 10-15ml Ficoll-Paque reagent (GE Lifesciences) as described by us before [40]. Samples were gently centrifuged for 40minutes at 400 x g maintaining 20°C temperature and without applying any brakes. A clear band of PBMCs was observed at the interface of Ficoll and plasma, and this band was collected. The collected PBMCs were washed with PBS and incubated at 37°C with red blood cell (RBC) lysis buffer. The cells were then centrifuged, washed with PBS and collected.

### Injection of PBMC in SCID mice

4-6 week old female SCID-NOD mice were obtained from Harlan laboratories and maintained under specific pathogen free condition. After a week of acclimatization in the new environment, 100μl PBS containing 40×10^6^ PBMCs were injected by intraperitoneal route in each mouse. After seven days of PBMCs injection, about 1×10^6^ U87MG cells in 1:1 mixture of PBS and matrigel were injected subcutaneously in both the flanks of SCID-NOD mice. Once tumor volume was 50-70mm^3^, mice were treated with 10mg/kg penfluridol by oral gavage everyday with 5 mice each in control and treatment group respectively.

### ELISA assay using tumor lysate

After terminating the experiment at day 40, tumors from SCID-NOD mice were removed from control and penfluridol treated groups. Tumors were lysed using protease and phosphatase inhibitors in PBS with subsequent vortexing and sonication. Protein was estimated using Bradford reagent (Bio-Rad). Equal amounts of protein from control and penfluridol treated tumor lysate samples were used to perform ELISA assay for CCL4 and IFNγ following the manufacturer's instructions (Affymetrix, ebioscience).

### Statistical analysis

Statistical analysis was performed using Prism 6.0 (GraphPad software Inc.). Tumor results were represented as means ± SEM. Data was analyzed by Student's t-test. Differences were considered statistically significant at p < 0.05 when compared with control.

## Abbreviations

PBMC: peripheral blood mononuclear cells
MDSC: myeloid derived suppressor cells
GBM: Glioblastoma multiforme
Treg: regulatory T cells
